# Volumetric Properties of the {*x*_1_[C_4_mim][MeSO_4_] + (1 − *x*_1_)MeOH} System at Temperatures from (283.15 to 333.15) K and Pressures from (0.1 to 35) MPa

**DOI:** 10.1007/s10953-013-0016-8

**Published:** 2013-05-25

**Authors:** Dobrochna Matkowska, Tadeusz Hofman

**Affiliations:** 1 Physical Chemistry Department, Faculty of Chemistry, Warsaw University of Technology, ul. Noakowskiego 3, 00-664 Warszawa, Poland; 2 Fire and Chemical Testing Department, Science and Research Centre for Fire Protection, ul. Nadwiślańska 213, 05-420 Józefów, Poland

**Keywords:** Ionic liquids, Density, Excess volume, Isothermal compressibility, Isobaric expansivity

## Abstract

Densities of pure 1-butyl-3-methylimidazolium methylsulfate, [C_4_mim][MeSO_4_], and its mixtures with methanol have been measured. Measurements were made with an accuracy of ±0.2 kg·m^−3^, using a vibrating-tube densimeter, over the temperature and pressure ranges (283.15–333.15) K and (0.1–35) MPa, respectively. The experimental densities for the pure ionic liquid and alcohol have been correlated by the Tait equation. The results for the {*x*_1_[C_4_mim][MeSO_4_] + (1 − *x*_1_)MeOH} system have been correlated by a van Laar equation involving parameters that are dependent on temperature and pressure. Excess volumes have been obtained directly from the experimental densities, while isobaric expansivities, isothermal compressibilities, and related excess properties were calculated from the correlation equation. Exceptionally strong pressure and temperature influences on these properties were observed.

**Electronic supplementary material:**

The online version of this article (doi:10.1007/s10953-013-0016-8) contains supplementary material, which is available to authorized users.

## Introduction

Ionic liquids (ILs) are compounds with growing interest because of their unique properties such as: negligible vapour pressure and excellent solvent power [1]. A large number of ILs are possible because they are the combination of large organic cations and inorganic or organic anions. To understand the nature of ILs and reasonably expand their possible applications, knowledge of their chemical and physical properties is required.

Thermodynamic properties of mixtures containing ILs and alcohols are very important from technological and theoretical points of view. The *p*,*ρ*,*T* properties of IL + organic solvents are among the most important thermodynamic properties. They provide very useful information on the intermolecular and structural interactions between the components of mixtures that have different shapes, sizes, and chemical nature. Previous studies have shown that addition of an alcohol into ILs significantly changes their phase behavior [2–4]. It is well known that even small additions of a low molar mass solvent can markedly increase or decrease the thermodynamic properties compared to the properties of the pure ILs [4]. Although ILs have been extensively studied, this study was undertaken because of the lack of experimental *p*,*ρ*,*T* data for IL + organic solvent systems and difficulties in understanding their peculiar properties.

Densities of pure [C_4_mim][MeSO_4_] as a function of temperature and under atmospheric pressure have been determined in a number of laboratories covering the temperature range (278.15–363.15) K [5–26]. As far as we know, we are the only group who has measured the [C_4_mim][MeSO_4_] density under high pressure (0.1–35) MPa [27].

There exist publications reporting excess volumes of the 1-butanol-3-methylimidazolium methylsulfate + methanol system [12, 20]. Both studies concern measurements under normal pressure and at temperatures of 298.15 K [12] and (298.15, 303.15, and 313.15) K [20]. The most extensively studied system is [C_4_mim][MeSO_4_] + ethanol [6, 7, 12, 15, 16, 20, 23]. Densities of multicomponent mixtures were obtained for systems consisting of 1-butanol-3-methylimidazolium methylsulfate and water [12, 16, 18, 26], propanol [20], 1-butanol [12], 1-hexanol [12], 1-octanol [12], 1-decanol [12], nitrometane [7, 8], 1,3-dichloropropane [7], ethylene glycol [7, 25], diethylene glycol monoethyl ether [7], and 1-butyl-3-methylimidazoliumtetrafluoroborate [5].

## Experimental Section

### Materials

The 1-butyl-3-methylimidazolium methylsulfate (Solvent Innovation Co., Köln, Germany) with mass fraction purity >99 %), and methanol (Aldrich, puriss >99.9 %), were dried and degassed as previously described [2]. After this procedure, the water content determined by Karl Fischer titration was about 200–400 ppm for the IL and about 20–100 ppm for the methanol.

The water used in the calibration was deionized and next degassed in the same manner as previously described [2]. The mixtures were prepared with a mole fraction uncertainty of about 10^−4^ using a balance with an accuracy of 5 × 10^−5^ g.

### Experimental Procedure and Apparatus

For the density measurements an Anton Paar vibrating-tube densimeter with measuring cell for high pressures and high temperatures (DMA 512P) and the mPDS 2000 evaluation unit were used. The density of a sample was determined by measuring the oscillation period of the U-shaped tube. The pressure was measured with a maximum uncertainty of ±0.01 MPa and the temperature was kept constant within ±0.01 K. A detailed description of the apparatus can be found in a previous article [28].

The densimeter was calibrated with an empty evacuated U-tube and water according to the procedure described by Lagourette et al. [29]. Water densities were calculated from the parameters proposed by the International Association for the properties of water and steam [30].

The combined expanded uncertainty for the measured densities of pure compounds should not exceed ±0.1 kg·m^−3^ at atmospheric pressure and about ±0.2 kg·m^−3^ at higher pressures. The combined standard uncertainty for the mixture densities is estimated to be ±0.05 kg·m^−3^.

## Results and Discussion

### Densities and Mechanical Coefficients of Pure 1-Butyl-3-methylimidazolium Methylsulfate and Methanol

**Fig. 1:**
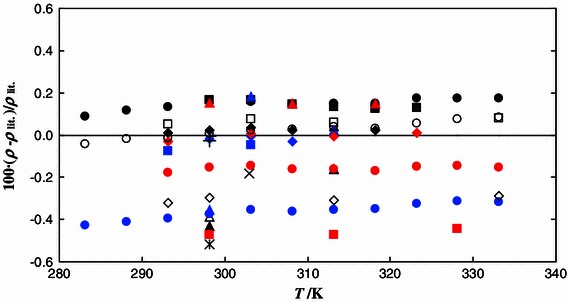
Fractional deviations 100(*ρ* − *ρ*_lit_)/*ρ*_lit_ between the measured and literature density values of [C_4_mim][MeSO_4_] under atmospheric pressures as a function of temperature. The literature data are taken from: *blue diamond* Navia et al. [5]; *black diamond* Garcia-Miaja et al. [6]; *dashed line* Iglesias-Otero et al. [7]; *filled square* Soriano et al. [9]; *square* Sánchez et al. [10], *white diamond* Tariq et al. [11]; *triangle* Domańska et al. [12]; *times* Kumełan et al. [13]; *blue circle* Pereiro et al. [14]; *red square* González et al. [16]; *red circle* Fernández et al. [17]; *filled circle* Singh and Kumar [19]; *blue triangle* Sibiya and Deenadayalu [20]; *asterisk* Shiflett et al. [21]; *black triangle* Torrecilla et al. [22]; *red diamond* Iglesias-Otero et al. [23]; *plus* Deenadayalu et al. [24]; *red triangle* Kumar et al. [25]; *blue square* Kumełan et al. [26]; *white circle* Matkowska and Hofman [27] (Color figure online)

**Fig. 2:**
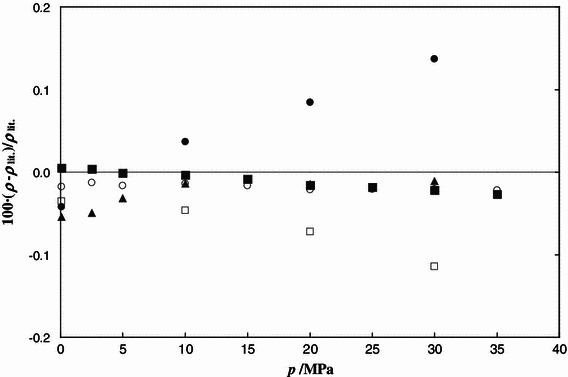
Fractional deviations 100(*ρ*–*ρ*_lit_)/*ρ*_lit_ between the measured and literature density values of methanol at 298.15 K as a function of pressure. The literature data are taken from: *circle* Gołdon et al. [28]; *black square* NIST Chemistry WebBook [31]; *black triangle* Machado and Streett [32]; *black circle* Ledwig and Würflinger [35]; *white square* Hrubỳ et al. [36]

Densities of the pure components, [C_4_mim][MeSO_4_] and MeOH, were measured at temperatures (283.15–343.15) K under pressures of (0.1–35.0) MPa. 198 experimental densities were obtained for both substances. A comparison between our results for this IL and the literature data measured under atmospheric pressure is shown in Fig. 1. Densities of [C_4_mim][MeSO_4_] presented in this work are about 0.2 % higher than the data reported by Soriano et al. [9], Singh and Kumar [19], Sibiya and Deenadayalu [20], and Kumar et al. [25] and are about 0.5 % lower than data reported by González et al. [16] and Shiflett et al. [21]. The deviations between densities for methanol measured in our laboratory and literature data are shown in Fig. 2. The maximum relative deviations do not exceed ±0.02 %. Good agreement with the densities calculated by the Gołdon et al. equation [28], the NIST correlation [31] and Machado and Streett [32] is noted.

Experimental densities of pure compounds were correlated by the Tait equation in the form

1 $$ \rho (T,p) = \frac{{\rho_{0} (T,p_{0} )}}{{1 - A\ln \frac{B(T) + p}{{B(T) + p_{0} }}}} $$

with the reference pressure *p*_0_ = 0.1 MPa. The *ρ*(*T*) and *B*(*T*) have the following functions of temperature

2 $$ \rho_{0} (T,p_{0} ) = \rho_{00} + \rho_{01} (T/K) + \rho_{02} (T/K)^{2} + \rho_{03} (T/K)^{3} $$

3 $$ B(T) = B_{0} + B_{1} (T/K) + B_{2} (T/K)^{2} $$

The number of terms in the above equations and constancy of the *A* parameter was determined by statistical analysis. The standard deviations and the fitted parameters of these equations are reported in Table 1.

**Table 1:** Coefficients of the Tait equations, Eqs. 1–3, fitted to the experimental densities of pure components [C_4_mim][MeSO_4_] and MeOH, and the root-mean-square deviations σ of the fit

*T*/K	*A* × 10^2^	*B* (MPa)	*ρ*_0_ (kg·m^−3^)	*σ* ^*a*^ (kg·m^−3^)
[C_4_mim]	8.50606	*B*_0_ (MPa) = 455.094	*ρ*_00_ (kg·m^−3^) = 1.39148 × 10^3^	0.011
[MeSO_4_]		*B*_1_ (MPa·K^−1^) = −0.72339	*ρ*_01_ (kg·m^−3^·K^−1^) = −0.58996	
			*ρ*_02_ (kg·m^−3^·K^−2^) = −9.13526 × 10^−5^	
MeOH	9.22363	*B*_0_ (MPa = 345.679	*ρ*_00_ (kg·m^−3^) = 1038.81	0.009
		*B*_1_ (MPa·K^−1^) = −1.30356	*ρ*_01_ (kg·m^−3^·K^−1^) = −7.57905	
		*B*_2_ (MPa·K^−2^) = 1.31682	*ρ*_02_ (kg·m^−3^·K^−2^) = −2.97068 × 10^−4^	

^a^ $$ \sigma = \left[ {\sum\limits_{i = 1}^{n} {\left( {\rho_{i}^{exp} - \rho_{i}^{\text{calc}} } \right)^{2} /n} } \right]^{1/2} $$

Equations 1–3 allowed us to calculate the related properties such as isothermal compressibility *κ* and isobaric expansivity *α* from the relations:

4 $$ \kappa = (\partial \ln \rho /\partial p)_{T} $$

and

5 $$ \alpha = - (\partial \ln \rho /\partial T)_{p} $$

**Fig. 3:**
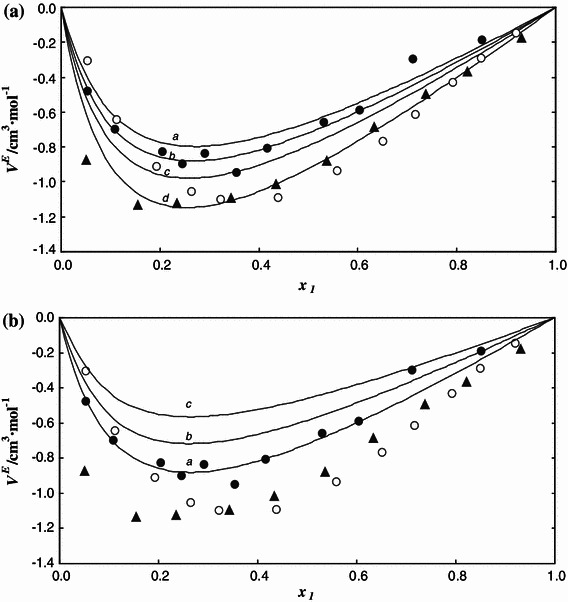
Excess volume *V* ^E^ of the {x_1_[C_4_mim][MeSO_4_] + (1 − *x*_1_)MeOH} system as a function of concentration *x*_1_ at **A** constant temperature *T* and pressure *p*/MPa = 0.1 and **b** constant pressure *p* and temperature *T*/K = 298.15. *Lines* are calculated from Eqs. 6–8 with the values of parameters given in Tables 1 and 2. Each line is labeled by a *letter* corresponding to **A** *T*/K: *a*, 283.15; *b*, 298.15; *c*, 313.15; *d*, 333.15; and **B** *p*/MPa: *a*, 0.1; *b*, 15; *c*, 35. Symbols denote experimental data: *filled circle* this work; *filled triangle* Domańska et al. [12]; *circle* Sibiya and Deenadayalu [20]; both measured at *p/*MPa = 0.1 and *T*/K = 298.15

**Fig. 4:**
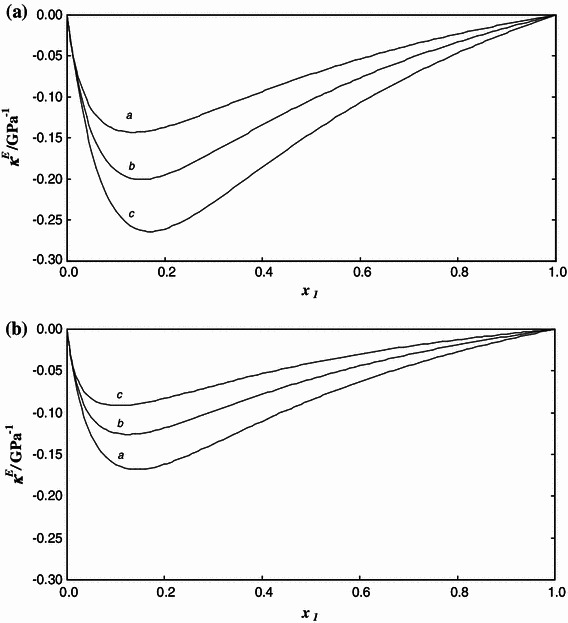
Excess isothermal compressibility *κ* ^E^ of the {*x*_1_[C_4_mim][MeSO_4_] + (1 − *x*_1_)MeOH} system as a function of concentration *x*_1_ at **A** constant temperature *T* and pressure *p*/MPa = 0.1 and **B** constant pressure *p* and temperature *T*/K = 298.15. *Lines* are calculated from Eqs. 6–8 with the parameter values given in Tables 1 and 2. Each line is labeled by a *letter* corresponding to **A** *T*/K: *a*, 283.15; *b*, 313.15; *c*, 333.15, and **B** *p*/MPa: *a*, 0.1; *b*, 15; *c*, 35

Figures 3 and 4 show calculated isothermal compressibility *κ* and isobaric expansivities α as a function of temperature and pressure for the IL and the alcohol. The temperature and pressure influences on the expansivities and compressibilities are much weaker for IL than for the alcohol. The calculated values of isobaric expansivities *α* for [C_4_mim][MeSO_4_] are between (4.96 and 5.49) × 10^−4^ K^−1^ while for methanol they are between (9.64 and 12.69) × 10^−4^ K^−1^ in the range of *p* = (0.1–35) MPa and *T* = (283.15–333.15) K. The values of isothermal compressibility *κ* for [C_4_mim][MeSO_4_] are between (0.30 and 0.40) GPa^−1^ while for methanol they are between (0.81 and 1.60) GPa^−1^ over the same pressure and temperature range. The isothermal compressibility slightly increases with increasing temperature and decreasing pressure.

The overall accuracies of the isothermal compressibility *κ* and isobaric expansivity *α* are difficult to estimate exactly as they depend on the form of the equation chosen to correlate experimental densities as a function of pressure and temperature. The maximum error of these derived parameters do not exceed ±0.01 GPa^−1^ for the isothermal compressibilities and ±0.1 × 10^−4^ K^−1^ for the isobaric expansivities.

### Densities and Excess Volumes of the 1-Butyl-3-methylimidazolium Methylsulfate + Methanol System

The densities for the {*x*_1_[C_4_mim][MeSO_4_] + (1 − *x*_1_)MeOH} system were measured at eleven different concentrations *x*_1_, at temperatures *T*/K = (283.15, 293.15, 298.15, 303.15, 313.15, 323.15, 333.15, 343.15) and under pressures *p*/MPa = (0.1, 2.5, 5.0, 10.0, 15.0, 20.0, 25.0, 30.0, 35.0). The IL + alcohol mixtures were prepared with an uncertainty in mole fraction estimated as being less than 5 × 10^−4^. We obtained 1089 data points excluding the densities of pure substances. These data, as well as the pure component data, are given in Table S1 in the Electronic Supplementary Material. The densities of the mixture were correlated by the following twelve-parameter equation in which the excess volume was formally represented by a sum of van Laar terms in which two such terms turned out to be suitable:

6 $$ \rho (T,p,x_{1} ) = \frac{{M_{1} x_{1} + M_{2} x_{2} }}{{M_{1} x_{1} /\rho_{1}^{0} (T,p) + M_{2} x_{2} /\rho_{2}^{0} (T,p) + x_{1} x_{2} \left( {\frac{{a_{0} \left( {T,p} \right)}}{{b_{0} \left( {T,p} \right)x_{1} + x_{2} }} + \frac{{a_{1} \left( {T,p} \right)}}{{b_{1} \left( {T,p} \right)x_{1} + x_{2} }}} \right)}} $$

The *a*_*i*_(*T,p*) and *b*_*i*_(*T,p*) coefficient depend linearly on *T* and *p* according to the formulae:

7 $$ [a_{i} (T,p)/{\text{cm}}^{3} \cdot {\text{mol}}^{ - 1} ] = a_{i0} + a_{i1} [(p/{\text{MPa}}) - 0.1] + a_{i2} [(T/{\text{K}}) - 283.15] $$

8 $$ b_{i} (T,p) = b_{i0} + b_{i1} [(p/{\text{MPa}}) - 0.1] + b_{i2} [(T/{\text{K}}) - 283.15] $$

It was confirmed statistically that higher-order terms in the above expansions could be disregarded. The equation requires twelve adjustable parameters that are given together with the standard deviation of the fit in Table 2.

**Table 2:** Coefficients of the equation fitted to the experimental densities of the {*x*_1_[C_4_mim][MeSO_4_] + (1 − *x*_1_) MeOH} system as a function of mole fraction concentration *x*_1_, temperature *T* and pressure *p*, Eqs. 6–8, and the root-mean-square deviations *σ* of the fit

*i*	*k*	*a*_*ik*_ (cm^3^·mol^−1^)	*b*_*ik*_	*σ* ^a^ (kg·m^−3^)
0	0	−11.7640	7.49182	0.58
0	1	7.74650 × 10^−2^	7.44898 × 10^−2^	
0	2	−4.27044 × 10^−2^	1.62784 × 10^−2^	
1	0	1.23238	17.1309	
1	1	0.29067	0.22478	
1	2	−0.10270	−0.14278	

^a^ $$ \sigma = \left[ {\sum\limits_{i = 1}^{n} {\left( {\rho_{i}^{ \exp } - \rho_{i}^{\text{calc}} } \right)^{2} /n} } \right]^{1/2} $$

Experimental excess volumes *V* ^E^ were calculated according to the formula

9 $$ V^{\text{E}} = M_{1} x_{1} (1/\rho - 1/\rho_{1}^{0} ) + M_{2} x_{2} (1/\rho - 1/\rho_{2}^{0} ) $$

where the *M*_*i*_ are molecular weights, *x*_*i*_ are concentrations, *ρ* designates densities of the solution and the $\rho_{i}^{0}$ are densities of pure *i*th components. The calculated excess volumes are presented in Table S2 in the Electronic Supplementary Material.

The excess volumes are reproduced by the following equation

10 $$ V^{\text{E}} (T,p,x_{1} ) = x_{1} x_{2} \left( {\frac{{a_{0} \left( {T,p} \right)}}{{b_{0} \left( {T,p} \right)x_{1} + x_{2} }} + \frac{{a_{1} \left( {T,p} \right)}}{{b_{1} \left( {T,p} \right)x_{1} + x_{2} }}} \right) $$

with the parameter values given in Table 2.

Figure 3 shows experimental and calculated excess volumes *V* ^E^ as a function of temperature and pressure. The excess volumes are negative and highly asymmetric with the minimum value at *x*_1_ = 0.28. They result from the differences among intermolecular interactions occurring in solution and in pure components. Among them, the attractive specific interactions (H-bonds) and repulsive ones (packing effects) are the most significant.

A relatively strong temperature and pressure influence on the values of excess volumes *V* ^E^ is observed. The excess volumes increase, i.e. become less negative, with decreases of the temperature and with increases of the pressure as was previously noted [2, 3, 28]. Since this former effect is strong it can be attributed to the presence of H-bonds. It is known that hydrogen bonding is more temperature-dependent and becomes negligible at high temperatures, compared with Coulombic interactions [33, 34]. However, the observed dependence cannot be simply and qualitatively explained as it results from the superposition of opposite effects including self-association of pure components and cross-association. The pressure influence on the excess volume is rather typical—increasing the pressure reduces differences in packing of the molecules.

Figure 3 also presents the data under atmospheric pressure and *T*/K = 298.15 K already reported in the literature [12, 20]. They are different than our data, the maximum deviation is ±50 % at high concentration of pure components and about 20 % lower at mole fraction *x*_1_ = 0.5. Also, by comparing differences of *V* ^E^ values for systems consisting of an IL and ethanol [6, 7, 12, 15, 16, 20, 23], we got the impression that such deviations are typical for these kinds of mixtures. It is worth noting that an increase of alcohol chain length resulted in an increase of the excess molar volumes.

### Mechanical Coefficients and the Corresponding Excess Properties of the 1-Butyl-3-methylimidazolium Methylsulfate + Methanol System

Equations 6–8 enable us to calculate isothermal compressibilities *κ* and isobaric expansivities *α* of the mixtures. The mechanical coefficients fall nonlinearly from a high value for pure methanol to the considerably lower one for pure IL. As the expansivities and compressibilities of an alcohol strongly depend on pressure and temperature, it is easy to predict that the {*x*_1_[C_4_mim][MeSO_4_] + (1 − *x*_1_)MeOH} system will still possess the above-mentioned relationship at alcohol concentrations higher than *x*_1_ = 0.35. Generally, the mechanical coefficients increase with increasing temperature and decreasing pressure.

The excess magnitudes *κ* ^E^ and *α* ^E^ were calculated by Eqs. 11 and 12, the $\varphi_{i}^{\text{id}}$ are ideal volume fractions given by Eq. 13, where $V_{i}^{0}$ represents the molar volume of a pure substance.

11 $$ \kappa^{\text{E}} = \kappa - \varphi_{1}^{\text{id}} \kappa_{1}^{0} - \varphi_{2}^{\text{id}} \kappa_{2}^{0} $$

12 $$ \alpha^{\text{E}} = \alpha - \varphi_{1}^{\text{id}} \alpha_{1}^{0} - \varphi_{2}^{\text{id}} \alpha_{2}^{0} $$

13 $$ \varphi_{i}^{\text{id}} = \frac{{x_{i} V_{i}^{0} }}{{x_{1} V_{1}^{0} + x_{2} V_{2}^{0} }} $$

**Fig. 5:**
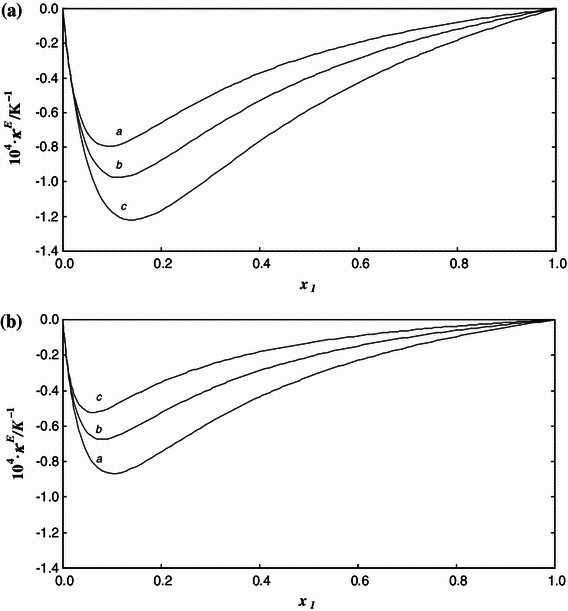
Excess isobaric expansivity *α* ^E^ of the {*x*_1_[C_4_mim][MeSO_4_] + (1 − *x*_1_)MeOHl} system as a function of concentration *x*_1_ at **A** constant temperature *T* and pressure *p*/MPa = 0.1, and **B** constant pressure *p* and temperature *T*/K = 298.15. *Lines* are calculated from Eqs. 6–8 with the parameter values given in Tables 1 and 2. Each line is labeled by a *letter* corresponding to **A** *T*/K: *a*, 283.15; *b*, 313.15; *c*, 333.15 and **B** *p*/MPa: *a*, 0.1; *b*, 15; *c*, 35

Figures 4 and 5 present the excess compressibilities and excess expansivities against mole fraction. The shape of the calculated curves is similar to those of previously measured systems consisting of an IL and methanol [3, 28]. The curves of excess magnitudes are unsymmetrical with the minimum located at a concentration of about *x*_1_ = 0.18, which is almost the same as for the {*x*_1_[C_2_mim][EtSO_4_] + (1 − *x*_1_)MeOH} system [3]. The absolute maximum values of both functions are lower than for the systems {*x*_1_[C_2_mim][EtSO_4_] + (1 − *x*_1_)MeOH} [3] and {*x*_1_[C_1_mim][MeSO_4_] + (1 − *x*_1_)MeOH} [28], although they are relatively large.

## Conclusions

Volumetric properties of pure 1-butyl-3-methylimidazolium methylsulfate and its mixtures with methanol show some interesting features that can be attributed to the presence of the ILs in the studied mixtures. Isobaric expansivities *α* and isothermal compressibilities *κ* of this IL have significantly lower values than for methanol. It was observed that the excess volumes *V* ^E^, excess isobaric expansivities *α* ^E^. and excess isothermal compressibilities *α* are significantly more dependent on temperature and pressure than for typical organic mixtures. The results from this study indicate that the negative *V* ^E^ values observed for the {*x*_1_[C_4_mim][MeSO_4_] + (1 − *x*_1_)MeOH} system can be explained by strong hydrogen bonding effects between molecules. An increase of temperature and decrease of pressure results in a decrease of the *V* ^E^ values.

## Electronic supplementary material

Below is the link to the electronic supplementary material.

Supplementary material 1 (DOCX 64 kb)
