# Supplementary material for: Volumetric Properties of the {x1[C4mim][MeSO4] + (1 − x1)MeOH} System at Temperatures from (283.15 to 333.15) K and Pressures from (0.1 to 35) MPa
Source: J Solution Chem. 2013 May 25;42(5):979–90. doi: 10.1007/s10953-013-0016-8 (PMC3676643; doi:10.1007/s10953-013-0016-8)
Supplement: Supplementary file 1 — Supplementary material 1 (DOCX 64 kb) [file 10953_2013_16_MOESM1_ESM.docx]

**Supplementary Material for:**

**Volumetric Properties of the {*x*_1_[C_4_mim][MeSO_4_] + (1 – *x*_1_)MeOH} System at Temperatures from (283.15 to 333.15) K and Pressures from (0.1 to 35) MPa**

**Dobrochna Matkowska · Tadeusz Hofman**

**Table S1** Experimental densities *ρ* for the {*x*_1_[C_4_mim][MeSO_4_] + (1 – *x*_1_)MeOH} system as a function of the mole fraction concentration *x*_1_, temperature *T*, and pressure *p*

|  | *ρ*/(kg·m^–3^) at *p*/MPa | | | | | | | | |
| --- | --- | --- | --- | --- | --- | --- | --- | --- | --- |
| *x*_1_ | 0.1 | 2.5 | 5 | 10 | 15 | 20 | 25 | 30 | 35 |
|  | *T* = 283.15 K | | | | | | | | |
| 0 | 800.36 | 802.51 | 804.68 | 808.86 | 812.88 | 816.71 | 820.40 | 823.96 | 827.40 |
| 0.05405 | 900.86 | 904.54 | 906.33 | 909.85 | 913.25 | 916.52 | 919.70 | 922.78 | 925.78 |
| 0.10968 | 972.36 | 974.18 | 975.52 | 978.64 | 981.67 | 984.60 | 987.44 | 990.22 | 992.94 |
| 0.20587 | 1048.76 | 1050.10 | 1051.49 | 1054.22 | 1056.86 | 1059.45 | 1061.96 | 1064.42 | 1066.87 |
| 0.24647 | 1072.67 | 1073.93 | 1075.23 | 1077.82 | 1080.34 | 1082.80 | 1085.20 | 1087.58 | 1089.90 |
| 0.29205 | 1092.87 | 1094.11 | 1095.39 | 1097.90 | 1100.36 | 1102.76 | 1105.09 | 1107.39 | 1109.67 |
| 0.35486 | 1118.07 | 1119.23 | 1120.45 | 1122.85 | 1125.19 | 1127.47 | 1129.72 | 1131.91 | 1134.09 |
| 0.41675 | 1135.71 | 1136.84 | 1138.00 | 1140.34 | 1142.61 | 1144.82 | 1146.99 | 1149.13 | 1151.25 |
| 0.53164 | 1161.78 | 1162.87 | 1163.96 | 1166.19 | 1168.36 | 1170.49 | 1172.58 | 1174.65 | 1176.66 |
| 0.60505 | 1175.15 | 1176.21 | 1177.31 | 1179.49 | 1181.64 | 1183.70 | 1185.76 | 1187.79 | 1189.79 |
| 0.71231 | 1189.52 | 1190.55 | 1191.62 | 1193.73 | 1195.82 | 1197.86 | 1199.86 | 1201.84 | 1203.80 |
| 0.85177 | 1205.05 | 1206.06 | 1207.12 | 1209.18 | 1211.20 | 1213.20 | 1215.15 | 1217.08 | 1219.00 |
| 1 | 1217.19 | 1218.18 | 1219.20 | 1221.22 | 1223.19 | 1225.14 | 1227.04 | 1228.94 | 1230.81 |
|  | *T* = 288.15 K | | | | | | | | |
| 0 | 795.79 | 797.99 | 800.20 | 804.51 | 808.46 | 812.45 | 816.33 | 819.94 | 823.46 |
| 0.05405 | 898.55 | 900.35 | 902.19 | 905.82 | 909.11 | 912.50 | 915.85 | 918.97 | 922.05 |
| 0.10968 | 968.32 | 969.90 | 971.53 | 974.72 | 977.64 | 980.66 | 983.68 | 986.50 | 989.26 |
| 0.20587 | 1044.98 | 1046.36 | 1047.76 | 1050.52 | 1053.07 | 1055.74 | 1058.42 | 1060.91 | 1063.38 |
| 0.24647 | 1068.72 | 1070.03 | 1071.36 | 1074.02 | 1076.41 | 1078.97 | 1081.53 | 1083.93 | 1086.30 |
| 0.29205 | 1089.42 | 1090.67 | 1091.95 | 1094.49 | 1096.82 | 1099.30 | 1101.78 | 1104.09 | 1106.40 |
| 0.35486 | 1114.47 | 1115.67 | 1116.89 | 1119.34 | 1121.53 | 1123.90 | 1126.28 | 1128.51 | 1130.73 |
| 0.41675 | 1132.11 | 1133.25 | 1134.43 | 1136.79 | 1138.92 | 1141.23 | 1143.55 | 1145.72 | 1147.88 |
| 0.53164 | 1158.14 | 1159.25 | 1160.38 | 1162.63 | 1164.68 | 1166.88 | 1169.12 | 1171.22 | 1173.29 |
| 0.60505 | 1171.69 | 1172.79 | 1173.87 | 1176.08 | 1178.08 | 1180.24 | 1182.43 | 1184.47 | 1186.49 |
| 0.71231 | 1185.99 | 1187.04 | 1188.12 | 1190.28 | 1192.24 | 1194.34 | 1196.48 | 1198.48 | 1200.48 |
| 0.85177 | 1201.62 | 1202.64 | 1203.69 | 1205.79 | 1207.68 | 1209.74 | 1211.85 | 1213.79 | 1215.75 |
| 1 | 1214.03 | 1215.03 | 1216.05 | 1218.09 | 1219.94 | 1221.95 | 1223.99 | 1225.90 | 1227.80 |
|  | *T* = 293.15 K | | | | | | | | |
| 0 | 791.17 | 793.44 | 795.72 | 800.14 | 804.33 | 808.35 | 812.21 | 815.93 | 819.53 |
| 0.05405 | 894.05 | 895.92 | 897.83 | 901.52 | 905.04 | 908.46 | 911.75 | 914.97 | 918.05 |
| 0.10968 | 964.15 | 965.78 | 967.44 | 970.70 | 973.83 | 976.89 | 979.82 | 982.72 | 985.53 |
| 0.20587 | 1041.08 | 1042.48 | 1043.93 | 1046.74 | 1049.48 | 1052.17 | 1054.75 | 1057.32 | 1059.81 |
| 0.24647 | 1064.77 | 1066.10 | 1067.47 | 1070.17 | 1072.78 | 1075.35 | 1077.83 | 1080.30 | 1082.71 |
| 0.29205 | 1085.50 | 1086.78 | 1088.11 | 1090.71 | 1093.23 | 1095.71 | 1098.10 | 1100.49 | 1102.82 |
| 0.35486 | 1110.91 | 1112.12 | 1113.37 | 1115.84 | 1118.23 | 1120.60 | 1122.89 | 1125.15 | 1127.39 |
| 0.41675 | 1128.33 | 1129.51 | 1130.72 | 1133.12 | 1135.45 | 1137.75 | 1139.99 | 1142.20 | 1144.39 |
| 0.53164 | 1154.40 | 1155.55 | 1156.70 | 1159.00 | 1161.23 | 1163.45 | 1165.57 | 1167.71 | 1169.80 |
| 0.60505 | 1168.07 | 1169.16 | 1170.31 | 1172.54 | 1174.71 | 1176.86 | 1178.95 | 1181.06 | 1183.09 |
| 0.71231 | 1182.30 | 1183.39 | 1184.50 | 1186.68 | 1188.80 | 1190.93 | 1192.96 | 1195.02 | 1197.02 |
| 0.85177 | 1198.01 | 1199.07 | 1200.14 | 1202.28 | 1204.36 | 1206.40 | 1208.42 | 1210.42 | 1212.35 |
| 1 | 1210.80 | 1211.77 | 1212.82 | 1214.88 | 1216.90 | 1218.90 | 1220.86 | 1222.80 | 1224.69 |
|  | *T* = 298.15 K | | | | | | | | |
| 0 | 786.37 | 788.71 | 791.05 | 795.59 | 799.89 | 803.98 | 807.93 | 811.71 | 815.34 |
| 0.05405 | 889.88 | 891.79 | 893.73 | 897.49 | 901.10 | 904.57 | 907.93 | 911.20 | 914.32 |
| 0.10968 | 960.14 | 961.96 | 963.65 | 966.95 | 970.25 | 973.29 | 976.30 | 979.22 | 982.05 |
| 0.20587 | 1037.46 | 1038.89 | 1040.34 | 1043.21 | 1045.99 | 1048.70 | 1051.35 | 1053.91 | 1056.43 |
| 0.24647 | 1061.15 | 1062.49 | 1063.89 | 1066.62 | 1069.27 | 1071.85 | 1074.40 | 1076.87 | 1079.27 |
| 0.29205 | 1081.94 | 1083.25 | 1084.58 | 1087.23 | 1089.79 | 1092.28 | 1094.75 | 1097.14 | 1099.49 |
| 0.35486 | 1107.32 | 1108.55 | 1109.81 | 1112.33 | 1114.76 | 1117.16 | 1119.50 | 1121.81 | 1124.09 |
| 0.41675 | 1125.11 | 1126.23 | 1127.45 | 1129.87 | 1132.21 | 1134.53 | 1136.80 | 1139.03 | 1141.18 |
| 0.53164 | 1151.34 | 1152.43 | 1153.60 | 1155.92 | 1158.15 | 1160.37 | 1162.53 | 1164.69 | 1166.75 |
| 0.60505 | 1164.67 | 1165.78 | 1166.92 | 1169.19 | 1171.40 | 1173.56 | 1175.71 | 1177.80 | 1179.85 |
| 0.71231 | 1178.95 | 1180.03 | 1181.14 | 1183.36 | 1185.51 | 1187.64 | 1189.73 | 1191.79 | 1193.79 |
| 0.85177 | 1194.97 | 1196.03 | 1197.10 | 1199.25 | 1201.34 | 1203.40 | 1205.44 | 1207.43 | 1209.38 |
| 1 | 1207.69 | 1208.71 | 1209.75 | 1211.84 | 1213.89 | 1215.90 | 1217.89 | 1219.84 | 1221.76 |
|  | *T* = 303.15 K | | | | | | | | |
| 0 | 781.65 | 784.04 | 786.44 | 791.07 | 795.46 | 799.62 | 803.79 | 807.65 | 811.37 |
| 0.05405 | 885.70 | 887.65 | 889.68 | 893.53 | 897.24 | 900.80 | 904.27 | 907.42 | 910.61 |
| 0.10968 | 956.26 | 957.95 | 959.68 | 963.03 | 966.28 | 969.42 | 972.43 | 975.40 | 978.27 |
| 0.20587 | 1033.73 | 1035.19 | 1036.68 | 1039.63 | 1042.50 | 1045.26 | 1047.95 | 1050.49 | 1052.96 |
| 0.24647 | 1057.55 | 1058.95 | 1060.38 | 1063.20 | 1065.97 | 1068.65 | 1071.28 | 1073.70 | 1076.04 |
| 0.29205 | 1078.43 | 1079.77 | 1081.15 | 1083.86 | 1086.47 | 1089.02 | 1091.53 | 1093.80 | 1096.17 |
| 0.35486 | 1103.89 | 1105.15 | 1106.45 | 1109.00 | 1111.47 | 1113.88 | 1116.27 | 1118.46 | 1120.73 |
| 0.41675 | 1121.43 | 1122.59 | 1123.83 | 1126.27 | 1128.65 | 1131.00 | 1133.31 | 1135.59 | 1137.79 |
| 0.53164 | 1147.59 | 1148.72 | 1149.90 | 1152.23 | 1154.50 | 1156.75 | 1159.08 | 1161.26 | 1163.40 |
| 0.60505 | 1161.36 | 1162.48 | 1163.63 | 1165.91 | 1168.16 | 1170.35 | 1172.51 | 1174.62 | 1176.70 |
| 0.71231 | 1175.81 | 1176.90 | 1178.04 | 1180.25 | 1182.43 | 1184.55 | 1186.63 | 1188.72 | 1190.72 |
| 0.85177 | 1191.50 | 1192.57 | 1193.67 | 1195.83 | 1197.95 | 1200.03 | 1202.06 | 1204.08 | 1206.06 |
| 1 | 1204.51 | 1205.57 | 1206.66 | 1208.84 | 1210.98 | 1213.11 | 1215.23 | 1217.20 | 1219.10 |
|  | *T* = 308.15 K | | | | | | | | |
| 0 | 777.11 | 779.58 | 782.07 | 786.83 | 791.38 | 795.67 | 799.81 | 803.75 | 807.55 |
| 0.05405 | 881.26 | 883.22 | 885.27 | 889.20 | 892.98 | 896.59 | 900.08 | 903.47 | 906.71 |
| 0.10968 | 952.03 | 953.75 | 955.53 | 958.98 | 962.29 | 965.49 | 968.61 | 971.64 | 974.55 |
| 0.20587 | 1029.79 | 1031.25 | 1032.77 | 1035.74 | 1038.62 | 1041.42 | 1044.14 | 1046.81 | 1049.41 |
| 0.24647 | 1053.70 | 1055.09 | 1056.53 | 1059.34 | 1062.08 | 1064.78 | 1067.36 | 1069.91 | 1072.40 |
| 0.29205 | 1074.70 | 1076.02 | 1077.41 | 1080.13 | 1082.77 | 1085.34 | 1087.85 | 1090.31 | 1092.72 |
| 0.35486 | 1100.07 | 1101.33 | 1102.65 | 1105.23 | 1107.75 | 1110.20 | 1112.60 | 1114.97 | 1117.28 |
| 0.41675 | 1117.69 | 1118.91 | 1120.18 | 1122.68 | 1125.10 | 1127.49 | 1129.82 | 1132.10 | 1134.35 |
| 0.53164 | 1144.07 | 1145.22 | 1146.44 | 1148.80 | 1151.13 | 1153.40 | 1155.64 | 1157.83 | 1159.97 |
| 0.60505 | 1157.81 | 1158.93 | 1160.10 | 1162.41 | 1164.69 | 1166.91 | 1169.09 | 1171.23 | 1173.34 |
| 0.71231 | 1172.16 | 1173.26 | 1174.41 | 1176.69 | 1178.89 | 1181.05 | 1183.19 | 1185.28 | 1187.35 |
| 0.85177 | 1187.88 | 1188.97 | 1190.09 | 1192.30 | 1194.46 | 1196.57 | 1198.66 | 1200.70 | 1202.72 |
| 1 | 1201.05 | 1202.09 | 1203.18 | 1205.32 | 1207.43 | 1209.50 | 1211.53 | 1213.53 | 1215.50 |
|  | *T* = 313.15 K | | | | | | | | |
| 0 | 772.35 | 774.88 | 777.44 | 782.36 | 786.99 | 791.40 | 795.62 | 799.67 | 803.52 |
| 0.05405 | 876.91 | 878.95 | 881.03 | 885.06 | 888.88 | 892.59 | 896.16 | 899.60 | 902.92 |
| 0.10968 | 948.08 | 949.82 | 951.61 | 955.12 | 958.47 | 961.74 | 964.90 | 967.97 | 970.97 |
| 0.20587 | 1026.05 | 1027.53 | 1029.06 | 1032.09 | 1034.99 | 1037.86 | 1040.62 | 1043.30 | 1045.93 |
| 0.24647 | 1050.02 | 1051.43 | 1052.89 | 1055.74 | 1058.53 | 1061.24 | 1063.88 | 1066.47 | 1068.99 |
| 0.29205 | 1070.93 | 1072.30 | 1073.69 | 1076.45 | 1079.13 | 1081.73 | 1084.30 | 1086.81 | 1089.25 |
| 0.35486 | 1096.48 | 1097.75 | 1099.10 | 1101.70 | 1104.24 | 1106.74 | 1109.18 | 1111.58 | 1113.91 |
| 0.41675 | 1114.17 | 1115.40 | 1116.66 | 1119.19 | 1121.62 | 1124.03 | 1126.40 | 1128.71 | 1131.00 |
| 0.53164 | 1140.64 | 1141.79 | 1143.00 | 1145.40 | 1147.73 | 1150.05 | 1152.31 | 1154.52 | 1156.71 |
| 0.60505 | 1154.21 | 1155.36 | 1156.55 | 1158.90 | 1161.18 | 1163.44 | 1165.67 | 1167.82 | 1169.96 |
| 0.71231 | 1168.77 | 1169.87 | 1171.03 | 1173.31 | 1175.55 | 1177.76 | 1179.91 | 1182.02 | 1184.11 |
| 0.85177 | 1184.59 | 1185.67 | 1186.78 | 1189.01 | 1191.51 | 1193.31 | 1195.41 | 1197.46 | 1199.50 |
| 1 | 1197.79 | 1198.85 | 1199.94 | 1202.11 | 1204.22 | 1206.31 | 1208.37 | 1210.40 | 1212.40 |
|  | *T* = 318.15 K | | | | | | | | |
| 0 | 767.51 | 770.15 | 772.79 | 777.85 | 782.62 | 787.14 | 791.47 | 795.59 | 799.53 |
| 0.05405 | 872.46 | 874.57 | 876.73 | 880.85 | 884.78 | 888.57 | 892.21 | 895.72 | 899.08 |
| 0.10968 | 943.79 | 945.60 | 947.45 | 951.04 | 954.49 | 957.84 | 961.07 | 964.18 | 967.22 |
| 0.20587 | 1022.07 | 1023.59 | 1025.19 | 1028.26 | 1031.24 | 1034.14 | 1036.96 | 1039.69 | 1042.35 |
| 0.24647 | 1046.10 | 1047.54 | 1049.05 | 1051.96 | 1054.81 | 1057.56 | 1060.26 | 1062.88 | 1065.46 |
| 0.29205 | 1067.14 | 1068.53 | 1069.97 | 1072.78 | 1075.51 | 1078.20 | 1080.80 | 1083.33 | 1085.82 |
| 0.35486 | 1092.77 | 1094.10 | 1095.45 | 1098.13 | 1100.73 | 1103.27 | 1105.75 | 1108.17 | 1110.55 |
| 0.41675 | 1110.29 | 1111.56 | 1112.89 | 1115.46 | 1117.97 | 1120.43 | 1122.84 | 1125.18 | 1127.49 |
| 0.53164 | 1136.85 | 1138.08 | 1139.29 | 1141.74 | 1144.14 | 1146.49 | 1148.79 | 1151.03 | 1153.23 |
| 0.60505 | 1150.68 | 1151.86 | 1153.09 | 1155.46 | 1157.81 | 1160.10 | 1162.36 | 1164.57 | 1166.72 |
| 0.71231 | 1165.23 | 1166.39 | 1167.57 | 1169.90 | 1172.17 | 1174.40 | 1176.60 | 1178.74 | 1180.85 |
| 0.85177 | 1180.89 | 1182.00 | 1183.17 | 1185.43 | 1187.64 | 1189.82 | 1191.96 | 1194.04 | 1196.11 |
| 1 | 1194.47 | 1195.54 | 1196.67 | 1198.87 | 1201.02 | 1203.16 | 1205.25 | 1207.30 | 1209.30 |
|  | *T* = 323.15 K | | | | | | | | |
| 0 | 763.04 | 765.72 | 768.42 | 773.60 | 778.47 | 783.08 | 787.49 | 791.71 | 795.73 |
| 0.05405 | 868.35 | 870.47 | 872.66 | 876.87 | 880.89 | 884.72 | 888.45 | 892.03 | 895.47 |
| 0.10968 | 939.94 | 941.76 | 943.64 | 947.29 | 950.80 | 954.19 | 957.48 | 960.67 | 963.76 |
| 0.20587 | 1018.41 | 1019.97 | 1021.56 | 1024.69 | 1027.71 | 1030.65 | 1033.50 | 1036.31 | 1039.02 |
| 0.24647 | 1042.42 | 1043.89 | 1045.41 | 1048.38 | 1051.27 | 1054.06 | 1056.78 | 1059.48 | 1062.07 |
| 0.29205 | 1063.66 | 1065.06 | 1066.51 | 1069.36 | 1072.13 | 1074.82 | 1077.48 | 1080.07 | 1082.59 |
| 0.35486 | 1089.44 | 1090.77 | 1092.14 | 1094.84 | 1097.46 | 1100.00 | 1102.52 | 1104.99 | 1107.41 |
| 0.41675 | 1106.82 | 1108.11 | 1109.42 | 1112.05 | 1114.59 | 1117.04 | 1119.51 | 1121.90 | 1124.24 |
| 0.53164 | 1133.67 | 1134.88 | 1136.14 | 1138.60 | 1141.03 | 1143.38 | 1145.69 | 1147.99 | 1150.21 |
| 0.60505 | 1147.53 | 1148.71 | 1149.91 | 1152.33 | 1154.68 | 1156.97 | 1159.26 | 1161.49 | 1163.68 |
| 0.71231 | 1162.20 | 1163.32 | 1164.51 | 1166.84 | 1169.13 | 1171.37 | 1173.59 | 1175.77 | 1177.90 |
| 0.85177 | 1177.79 | 1178.89 | 1180.05 | 1182.33 | 1184.56 | 1186.74 | 1188.90 | 1191.04 | 1193.12 |
| 1 | 1191.45 | 1192.52 | 1193.64 | 1195.88 | 1198.04 | 1200.18 | 1202.29 | 1204.38 | 1206.40 |
|  | *T* = 328.15 K | | | | | | | | |
| 0 | 758.09 | 760.88 | 763.69 | 768.98 | 773.98 | 778.75 | 783.26 | 787.56 | 791.66 |
| 0.05405 | 863.85 | 866.06 | 868.31 | 872.60 | 876.71 | 880.67 | 884.46 | 888.10 | 891.60 |
| 0.10968 | 935.74 | 937.63 | 939.54 | 943.28 | 946.86 | 950.34 | 953.68 | 956.93 | 960.04 |
| 0.20587 | 1014.58 | 1016.16 | 1017.82 | 1020.98 | 1024.07 | 1027.10 | 1029.99 | 1032.83 | 1035.57 |
| 0.24647 | 1038.66 | 1040.18 | 1041.72 | 1044.73 | 1047.68 | 1050.55 | 1053.33 | 1056.02 | 1058.65 |
| 0.29205 | 1060.11 | 1061.54 | 1063.04 | 1065.92 | 1068.74 | 1071.49 | 1074.16 | 1076.78 | 1079.33 |
| 0.35486 | 1085.78 | 1087.14 | 1088.54 | 1091.27 | 1093.96 | 1096.56 | 1099.11 | 1101.62 | 1104.03 |
| 0.41675 | 1103.48 | 1104.80 | 1106.15 | 1108.76 | 1111.36 | 1113.88 | 1116.34 | 1118.76 | 1121.10 |
| 0.53164 | 1130.11 | 1131.35 | 1132.62 | 1135.13 | 1137.58 | 1140.01 | 1142.35 | 1144.66 | 1146.92 |
| 0.60505 | 1144.05 | 1145.25 | 1146.50 | 1148.93 | 1151.33 | 1153.70 | 1156.01 | 1158.26 | 1160.48 |
| 0.71231 | 1158.63 | 1159.82 | 1161.08 | 1163.44 | 1165.76 | 1168.06 | 1170.31 | 1172.51 | 1174.67 |
| 0.85177 | 1174.44 | 1175.59 | 1176.76 | 1179.06 | 1181.32 | 1183.58 | 1185.75 | 1187.90 | 1190.00 |
| 1 | 1188.27 | 1189.38 | 1190.51 | 1192.74 | 1194.95 | 1197.14 | 1199.27 | 1201.37 | 1203.42 |
|  | *T* = 333.15 K | | | | | | | | |
| 0 | 753.23 | 756.11 | 759.01 | 764.45 | 769.64 | 774.48 | 779.10 | 783.50 | 787.69 |
| 0.05405 | 859.42 | 861.68 | 863.98 | 868.37 | 872.61 | 876.65 | 880.52 | 884.23 | 887.80 |
| 0.10968 | 931.54 | 933.46 | 935.44 | 939.24 | 942.94 | 946.46 | 949.87 | 953.18 | 956.37 |
| 0.20587 | 1010.82 | 1012.44 | 1014.10 | 1017.33 | 1020.50 | 1023.56 | 1026.50 | 1029.36 | 1032.16 |
| 0.24647 | 1035.02 | 1036.56 | 1038.14 | 1041.20 | 1044.21 | 1047.10 | 1049.93 | 1052.70 | 1055.36 |
| 0.29205 | 1056.27 | 1057.75 | 1059.25 | 1062.20 | 1065.08 | 1067.87 | 1070.60 | 1073.25 | 1075.83 |
| 0.35486 | 1082.05 | 1083.47 | 1084.89 | 1087.65 | 1090.40 | 1093.04 | 1095.63 | 1098.17 | 1100.63 |
| 0.41675 | 1099.95 | 1101.30 | 1102.68 | 1105.36 | 1107.99 | 1110.54 | 1113.04 | 1115.49 | 1117.87 |
| 0.53164 | 1126.47 | 1127.76 | 1129.04 | 1131.59 | 1134.11 | 1136.55 | 1138.93 | 1141.28 | 1143.57 |
| 0.60505 | 1140.67 | 1141.88 | 1143.15 | 1145.62 | 1148.07 | 1150.45 | 1152.77 | 1155.08 | 1157.32 |
| 0.71231 | 1155.20 | 1156.41 | 1157.65 | 1160.05 | 1162.44 | 1164.73 | 1167.02 | 1169.27 | 1171.43 |
| 0.85177 | 1171.23 | 1172.38 | 1173.58 | 1175.91 | 1178.21 | 1180.47 | 1182.67 | 1184.84 | 1186.98 |
| 1 | 1184.96 | 1186.08 | 1187.26 | 1189.52 | 1191.79 | 1194.10 | 1196.14 | 1198.27 | 1200.35 |

**Table S2** Experimental excess volumes *V*^E^ for {*x*_1_[C_4_mim][MeSO_4_] + (1 – *x*_1_)MeOH} solutions as a function of mole fraction concentration *x*_1_, temperature *T*, and pressure *p*

|  | *V*^E^/cm^3^⋅mol^–1^ at *p*/MPa | | | | | | | | |
| --- | --- | --- | --- | --- | --- | --- | --- | --- | --- |
|  | 0.1 | 2.5 | 5 | 10 | 15 | 20 | 25 | 30 | 35 |
| *x*_1_ | *T* = 283.15 K | | | | | | | | |
| 0.05405 | –0.32 | –0.41 | –0.39 | –0.37 | –0.34 | –0.32 | –0.30 | –0.29 | –0.27 |
| 0.10968 | –0.63 | –0.62 | –0.58 | –0.55 | –0.51 | –0.48 | –0.45 | –0.43 | –0.40 |
| 0.20587 | –0.73 | –0.70 | –0.68 | –0.64 | –0.60 | –0.56 | –0.53 | –0.50 | –0.47 |
| 0.24647 | –0.83 | –0.80 | –0.77 | –0.73 | –0.68 | –0.65 | –0.61 | –0.58 | –0.55 |
| 0.29205 | –0.75 | –0.73 | –0.70 | –0.66 | –0.62 | –0.58 | –0.55 | –0.52 | –0.49 |
| 0.35486 | –0.87 | –0.84 | –0.82 | –0.78 | –0.73 | –0.70 | –0.66 | –0.63 | –0.60 |
| 0.41675 | –0.75 | –0.72 | –0.70 | –0.66 | –0.62 | –0.58 | –0.55 | –0.52 | –0.50 |
| 0.53164 | –0.62 | –0.60 | –0.58 | –0.54 | –0.51 | –0.48 | –0.46 | –0.43 | –0.41 |
| 0.60505 | –0.59 | –0.57 | –0.56 | –0.53 | –0.51 | –0.48 | –0.46 | –0.44 | –0.42 |
| 0.71231 | –0.36 | –0.35 | –0.34 | –0.31 | –0.29 | –0.28 | –0.26 | –0.25 | –0.23 |
| 0.85177 | –0.23 | –0.22 | –0.22 | –0.21 | –0.20 | –0.19 | –0.18 | –0.17 | –0.17 |
|  | *T* = 288.15 K | | | | | | | | |
| 0.05405 | –0.44 | –0.43 | –0.41 | –0.38 | –0.36 | –0.34 | –0.32 | –0.30 | –0.28 |
| 0.10968 | –0.65 | –0.63 | –0.60 | –0.56 | –0.53 | –0.50 | –0.47 | –0.44 | –0.41 |
| 0.20587 | –0.76 | –0.73 | –0.71 | –0.66 | –0.62 | –0.58 | –0.54 | –0.51 | –0.48 |
| 0.24647 | –0.84 | –0.81 | –0.79 | –0.74 | –0.69 | –0.65 | –0.61 | –0.58 | –0.55 |
| 0.29205 | –0.80 | –0.77 | –0.74 | –0.69 | –0.65 | –0.61 | –0.57 | –0.54 | –0.51 |
| 0.35486 | –0.89 | –0.86 | –0.84 | –0.79 | –0.75 | –0.71 | –0.67 | –0.64 | –0.61 |
| 0.41675 | –0.76 | –0.73 | –0.71 | –0.67 | –0.62 | –0.59 | –0.56 | –0.53 | –0.50 |
| 0.53164 | –0.61 | –0.59 | –0.57 | –0.54 | –0.50 | –0.47 | –0.44 | –0.42 | –0.40 |
| 0.60505 | –0.59 | –0.58 | –0.56 | –0.53 | –0.50 | –0.47 | –0.45 | –0.43 | –0.41 |
| 0.71231 | –0.34 | –0.33 | –0.31 | –0.29 | –0.28 | –0.26 | –0.24 | –0.22 | –0.21 |
| 0.85177 | –0.20 | –0.19 | –0.19 | –0.18 | –0.17 | –0.16 | –0.15 | –0.14 | –0.14 |
|  | *T* = 293.15 K | | | | | | | | |
| 0.05405 | –0.45 | –0.43 | –0.42 | –0.39 | –0.36 | –0.34 | –0.32 | –0.30 | –0.28 |
| 0.10968 | –0.67 | –0.65 | –0.62 | –0.58 | –0.54 | –0.51 | –0.48 | –0.45 | –0.42 |
| 0.20587 | –0.78 | –0.76 | –0.73 | –0.68 | –0.63 | –0.59 | –0.56 | –0.52 | –0.49 |
| 0.24647 | –0.85 | –0.83 | –0.80 | –0.75 | –0.70 | –0.66 | –0.62 | –0.59 | –0.56 |
| 0.29205 | –0.81 | –0.78 | –0.75 | –0.70 | –0.66 | –0.62 | –0.58 | –0.54 | –0.51 |
| 0.35486 | –0.92 | –0.90 | –0.87 | –0.82 | –0.77 | –0.73 | –0.69 | –0.66 | –0.63 |
| 0.41675 | –0.76 | –0.74 | –0.71 | –0.67 | –0.63 | –0.59 | –0.56 | –0.52 | –0.50 |
| 0.53164 | –0.60 | –0.58 | –0.56 | –0.53 | –0.49 | –0.46 | –0.43 | –0.41 | –0.39 |
| 0.60505 | –0.59 | –0.57 | –0.55 | –0.52 | –0.49 | –0.46 | –0.44 | –0.42 | –0.40 |
| 0.71231 | –0.30 | –0.30 | –0.29 | –0.27 | –0.24 | –0.23 | –0.21 | –0.19 | –0.18 |
| 0.85177 | –0.16 | –0.16 | –0.16 | –0.15 | –0.14 | –0.13 | –0.12 | –0.11 | –0.11 |
|  | *T* = 298.15 K | | | | | | | | |
| 0.05405 | –0.48 | –0.46 | –0.45 | –0.41 | –0.39 | –0.36 | –0.34 | –0.32 | –0.30 |
| 0.10968 | –0.70 | –0.69 | –0.66 | –0.62 | –0.58 | –0.54 | –0.51 | –0.48 | –0.45 |
| 0.20587 | –0.83 | –0.80 | –0.77 | –0.72 | –0.67 | –0.63 | –0.59 | –0.55 | –0.52 |
| 0.24647 | –0.90 | –0.86 | –0.83 | –0.78 | –0.73 | –0.69 | –0.65 | –0.61 | –0.57 |
| 0.29205 | –0.84 | –0.81 | –0.79 | –0.73 | –0.69 | –0.64 | –0.60 | –0.57 | –0.54 |
| 0.35486 | –0.95 | –0.92 | –0.89 | –0.84 | –0.79 | –0.75 | –0.71 | –0.68 | –0.65 |
| 0.41675 | –0.81 | –0.78 | –0.75 | –0.70 | –0.66 | –0.62 | –0.58 | –0.55 | –0.52 |
| 0.53164 | –0.66 | –0.63 | –0.61 | –0.57 | –0.53 | –0.50 | –0.46 | –0.44 | –0.41 |
| 0.60505 | –0.59 | –0.58 | –0.56 | –0.52 | –0.49 | –0.46 | –0.44 | –0.41 | –0.39 |
| 0.71231 | –0.30 | –0.29 | –0.28 | –0.26 | –0.23 | –0.21 | –0.20 | –0.18 | –0.17 |
| 0.85177 | –0.19 | –0.18 | –0.17 | –0.16 | –0.15 | –0.14 | –0.13 | –0.12 | –0.11 |
|  | *T* = 303.15 K | | | | | | | | |
| 0.05405 | –0.51 | –0.49 | –0.48 | –0.44 | –0.41 | –0.39 | –0.36 | –0.33 | –0.31 |
| 0.10968 | –0.75 | –0.72 | –0.69 | –0.64 | –0.60 | –0.56 | –0.51 | –0.48 | –0.45 |
| 0.20587 | –0.87 | –0.84 | –0.81 | –0.75 | –0.70 | –0.66 | –0.61 | –0.56 | –0.53 |
| 0.24647 | –0.94 | –0.91 | –0.88 | –0.82 | –0.77 | –0.73 | –0.68 | –0.63 | –0.59 |
| 0.29205 | –0.89 | –0.86 | –0.83 | –0.77 | –0.72 | –0.67 | –0.62 | –0.57 | –0.54 |
| 0.35486 | –1.00 | –0.96 | –0.94 | –0.88 | –0.82 | –0.77 | –0.72 | –0.67 | –0.64 |
| 0.41675 | –0.83 | –0.79 | –0.76 | –0.70 | –0.65 | –0.60 | –0.56 | –0.52 | –0.49 |
| 0.53164 | –0.64 | –0.61 | –0.59 | –0.54 | –0.49 | –0.45 | –0.42 | –0.39 | –0.37 |
| 0.60505 | –0.62 | –0.60 | –0.57 | –0.53 | –0.49 | –0.45 | –0.41 | –0.39 | –0.36 |
| 0.71231 | –0.34 | –0.32 | –0.31 | –0.27 | –0.24 | –0.20 | –0.16 | –0.15 | –0.13 |
| 0.85177 | –0.16 | –0.15 | –0.14 | –0.11 | –0.09 | –0.07 | –0.04 | –0.03 | –0.03 |
|  | *T* = 308.15 K | | | | | | | | |
| 0.05405 | –0.52 | –0.50 | –0.48 | –0.44 | –0.41 | –0.38 | –0.36 | –0.34 | –0.31 |
| 0.10968 | –0.77 | –0.74 | –0.71 | –0.66 | –0.61 | –0.57 | –0.53 | –0.50 | –0.47 |
| 0.20587 | –0.90 | –0.86 | –0.83 | –0.77 | –0.72 | –0.67 | –0.63 | –0.59 | –0.55 |
| 0.24647 | –0.97 | –0.94 | –0.90 | –0.84 | –0.78 | –0.74 | –0.69 | –0.65 | –0.61 |
| 0.29205 | –0.93 | –0.89 | –0.86 | –0.80 | –0.74 | –0.69 | –0.65 | –0.61 | –0.57 |
| 0.35486 | –1.02 | –0.99 | –0.95 | –0.89 | –0.84 | –0.79 | –0.75 | –0.71 | –0.67 |
| 0.41675 | –0.85 | –0.82 | –0.79 | –0.73 | –0.68 | –0.64 | –0.60 | –0.56 | –0.53 |
| 0.53164 | –0.68 | –0.65 | –0.63 | –0.58 | –0.54 | –0.50 | –0.47 | –0.43 | –0.40 |
| 0.60505 | –0.65 | –0.62 | –0.60 | –0.56 | –0.52 | –0.49 | –0.46 | –0.43 | –0.41 |
| 0.71231 | –0.34 | –0.32 | –0.31 | –0.28 | –0.25 | –0.23 | –0.21 | –0.19 | –0.17 |
| 0.85177 | –0.15 | –0.14 | –0.13 | –0.12 | –0.11 | –0.10 | –0.09 | –0.08 | –0.07 |
|  | *T* = 313.15 K | | | | | | | | |
| 0.05405 | –0.55 | –0.52 | –0.50 | –0.46 | –0.43 | –0.40 | –0.37 | –0.35 | –0.33 |
| 0.10968 | –0.81 | –0.78 | –0.75 | –0.69 | –0.64 | –0.60 | –0.56 | –0.52 | –0.49 |
| 0.20587 | –0.94 | –0.91 | –0.87 | –0.81 | –0.75 | –0.70 | –0.66 | –0.61 | –0.57 |
| 0.24647 | –1.02 | –0.98 | –0.94 | –0.88 | –0.82 | –0.77 | –0.72 | –0.68 | –0.64 |
| 0.29205 | –0.96 | –0.92 | –0.89 | –0.82 | –0.77 | –0.71 | –0.67 | –0.63 | –0.59 |
| 0.35486 | –1.06 | –1.02 | –0.99 | –0.92 | –0.87 | –0.82 | –0.77 | –0.73 | –0.69 |
| 0.41675 | –0.89 | –0.85 | –0.82 | –0.76 | –0.71 | –0.66 | –0.61 | –0.57 | –0.54 |
| 0.53164 | –0.71 | –0.68 | –0.65 | –0.60 | –0.55 | –0.52 | –0.48 | –0.45 | –0.42 |
| 0.60505 | –0.65 | –0.62 | –0.60 | –0.56 | –0.52 | –0.49 | –0.46 | –0.43 | –0.40 |
| 0.71231 | –0.35 | –0.33 | –0.32 | –0.29 | –0.26 | –0.24 | –0.21 | –0.19 | –0.17 |
| 0.85177 | –0.16 | –0.15 | –0.14 | –0.12 | –0.16 | –0.10 | –0.08 | –0.07 | –0.06 |
|  | *T* = 318.15 K | | | | | | | | |
| 0.05405 | –0.57 | –0.55 | –0.52 | –0.48 | –0.45 | –0.41 | –0.39 | –0.36 | –0.34 |
| 0.10968 | –0.84 | –0.80 | –0.77 | –0.71 | –0.66 | –0.61 | –0.57 | –0.54 | –0.50 |
| 0.20587 | –0.98 | –0.94 | –0.90 | –0.83 | –0.77 | –0.72 | –0.67 | –0.63 | –0.59 |
| 0.24647 | –1.05 | –1.01 | –0.97 | –0.90 | –0.84 | –0.78 | –0.73 | –0.69 | –0.65 |
| 0.29205 | –1.00 | –0.96 | –0.92 | –0.85 | –0.79 | –0.74 | –0.69 | –0.64 | –0.61 |
| 0.35486 | –1.10 | –1.06 | –1.02 | –0.95 | –0.90 | –0.84 | –0.79 | –0.75 | –0.71 |
| 0.41675 | –0.90 | –0.86 | –0.83 | –0.77 | –0.71 | –0.66 | –0.62 | –0.57 | –0.54 |
| 0.53164 | –0.70 | –0.68 | –0.65 | –0.59 | –0.55 | –0.51 | –0.47 | –0.44 | –0.41 |
| 0.60505 | –0.67 | –0.64 | –0.62 | –0.57 | –0.53 | –0.50 | –0.46 | –0.44 | –0.41 |
| 0.71231 | –0.35 | –0.34 | –0.32 | –0.29 | –0.26 | –0.23 | –0.21 | –0.19 | –0.17 |
| 0.85177 | –0.11 | –0.11 | –0.10 | –0.08 | –0.07 | –0.06 | –0.04 | –0.03 | –0.03 |
|  | *T* = 323.15 K | | | | | | | | |
| 0.05405 | –0.59 | –0.57 | –0.54 | –0.50 | –0.46 | –0.43 | –0.40 | –0.37 | –0.35 |
| 0.10968 | –0.87 | –0.83 | –0.80 | –0.74 | –0.68 | –0.64 | –0.59 | –0.55 | –0.52 |
| 0.20587 | –1.01 | –0.97 | –0.93 | –0.86 | –0.80 | –0.74 | –0.69 | –0.65 | –0.61 |
| 0.24647 | –1.08 | –1.04 | –1.00 | –0.92 | –0.86 | –0.80 | –0.75 | –0.70 | –0.66 |
| 0.29205 | –1.03 | –0.99 | –0.95 | –0.88 | –0.81 | –0.76 | –0.71 | –0.66 | –0.62 |
| 0.35486 | –1.14 | –1.10 | –1.06 | –0.98 | –0.92 | –0.86 | –0.81 | –0.77 | –0.73 |
| 0.41675 | –0.91 | –0.88 | –0.84 | –0.78 | –0.72 | –0.67 | –0.62 | –0.58 | –0.54 |
| 0.53164 | –0.74 | –0.71 | –0.68 | –0.62 | –0.57 | –0.53 | –0.49 | –0.46 | –0.42 |
| 0.60505 | –0.69 | –0.67 | –0.64 | –0.59 | –0.55 | –0.51 | –0.48 | –0.45 | –0.42 |
| 0.71231 | –0.38 | –0.36 | –0.34 | –0.30 | –0.28 | –0.25 | –0.23 | –0.20 | –0.18 |
| 0.85177 | –0.12 | –0.11 | –0.10 | –0.08 | –0.07 | –0.05 | –0.04 | –0.03 | –0.02 |
|  | *T* = 328.15 K | | | | | | | | |
| 0.05405 | –0.62 | –0.59 | –0.56 | –0.52 | –0.48 | –0.44 | –0.41 | –0.38 | –0.36 |
| 0.10968 | –0.91 | –0.87 | –0.83 | –0.77 | –0.71 | –0.66 | –0.61 | –0.57 | –0.54 |
| 0.20587 | –1.06 | –1.02 | –0.97 | –0.90 | –0.83 | –0.77 | –0.72 | –0.67 | –0.63 |
| 0.24647 | –1.13 | –1.08 | –1.04 | –0.96 | –0.89 | –0.83 | –0.78 | –0.72 | –0.68 |
| 0.29205 | –1.09 | –1.04 | –1.00 | –0.93 | –0.86 | –0.80 | –0.74 | –0.69 | –0.65 |
| 0.35486 | –1.17 | –1.13 | –1.09 | –1.01 | –0.95 | –0.89 | –0.83 | –0.79 | –0.74 |
| 0.41675 | –0.97 | –0.93 | –0.89 | –0.82 | –0.76 | –0.71 | –0.66 | –0.61 | –0.57 |
| 0.53164 | –0.75 | –0.72 | –0.69 | –0.63 | –0.58 | –0.54 | –0.49 | –0.46 | –0.42 |
| 0.60505 | –0.70 | –0.68 | –0.65 | –0.60 | –0.56 | –0.52 | –0.48 | –0.45 | –0.42 |
| 0.71231 | –0.36 | –0.35 | –0.33 | –0.30 | –0.27 | –0.24 | –0.21 | –0.19 | –0.17 |
| 0.85177 | –0.11 | –0.10 | –0.09 | –0.07 | –0.06 | –0.05 | –0.03 | –0.02 | –0.01 |
|  | *T* = 333.15 K | | | | | | | | |
| 0.05405 | –0.65 | –0.62 | –0.59 | –0.54 | –0.50 | –0.46 | –0.43 | –0.40 | –0.37 |
| 0.10968 | –0.95 | –0.90 | –0.86 | –0.79 | –0.73 | –0.68 | –0.63 | –0.59 | –0.55 |
| 0.20587 | –1.12 | –1.07 | –1.02 | –0.94 | –0.87 | –0.80 | –0.75 | –0.70 | –0.66 |
| 0.24647 | –1.19 | –1.14 | –1.09 | –1.01 | –0.93 | –0.86 | –0.81 | –0.76 | –0.71 |
| 0.29205 | –1.12 | –1.08 | –1.03 | –0.95 | –0.88 | –0.81 | –0.76 | –0.71 | –0.66 |
| 0.35486 | –1.21 | –1.17 | –1.12 | –1.04 | –0.97 | –0.90 | –0.85 | –0.80 | –0.76 |
| 0.41675 | –1.02 | –0.98 | –0.93 | –0.86 | –0.80 | –0.73 | –0.69 | –0.64 | –0.59 |
| 0.53164 | –0.77 | –0.74 | –0.70 | –0.64 | –0.59 | –0.53 | –0.50 | –0.46 | –0.42 |
| 0.60505 | –0.74 | –0.71 | –0.68 | –0.63 | –0.58 | –0.53 | –0.50 | –0.47 | –0.44 |
| 0.71231 | –0.38 | –0.36 | –0.34 | –0.30 | –0.27 | –0.22 | –0.21 | –0.19 | –0.17 |
| 0.85177 | –0.14 | –0.13 | –0.12 | –0.10 | –0.08 | –0.05 | –0.05 | –0.04 | –0.03 |
